# Loss to Followup in HIV-Infected Patients from Asia-Pacific Region: Results from TAHOD

**DOI:** 10.1155/2012/375217

**Published:** 2012-02-22

**Authors:** Jialun Zhou, Junko Tanuma, Romanee Chaiwarith, Christopher K. C. Lee, Matthew G. Law, Nagalingeswaran Kumarasamy, Praphan Phanuphak, Yi-Ming A. Chen, Sasisopin Kiertiburanakul, Fujie Zhang, Saphonn Vonthanak, Rossana Ditangco, Sanjay Pujari, Jun Yong Choi, Tuti Parwati Merati, Evy Yunihastuti, Patrick C. K. Li, Adeeba Kamarulzaman, Van Kinh Nguyen, Thi Thanh Thuy Pham, Poh Lian Lim

**Affiliations:** ^1^The Kirby Institute, The University of New South Wales, Sydney, NSW 2034, Australia; ^2^National Center for Global Health and Medicine, Tokyo 162-8655, Japan; ^3^Research Institute for Health Sciences, Chiang Mai 50200, Thailand; ^4^Department of Medicine, Hospital Sungai Buloh, 47000 Kuala Lumpur, Malaysia; ^5^YRG Centre for AIDS Research and Education, Chennai 600113, India; ^6^HIV-NAT/Thai Red Cross AIDS Research Centre, Bangkok 10330, Thailand; ^7^Taipei Veterans General Hospital and AIDS Prevention and Research Centre, National Yang-Ming University, Taipei 112, Taiwan; ^8^Ramathibodi Hospital, Faculty of Medicine, Mahidol University, Bangkok 10400, Thailand; ^9^Beijing Ditan Hospital, Capital Medical University, Beijing 100050, China; ^10^National Center for HIV/AIDS, Dermatology and STDs, Phnom Penh, Cambodia; ^11^Research Institute for Tropical Medicine, 1781 Manila, Philippines; ^12^Institute of Infectious Diseases, Pune 411037, India; ^13^Division of Infectious Diseases, Department of Internal Medicine, Yonsei University College of Medicine, Seoul 120-752, Republic of Korea; ^14^Faculty of Medicine, Udayana University and Sanglah Hospital, Bali 80233, Indonesia; ^15^Working Group on AIDS, Faculty of Medicine, University of Indonesia/Ciptomangunkusumo Hospital, Jakarta 10430, Indonesia; ^16^Department of Medicine, Queen Elizabeth Hospital, Hong Kong; ^17^Faculty of Medicine, University of Malaya Medical Centre, 59100 Kuala Lumpur, Malaysia; ^18^National Hospital for Tropical Diseases, Hanoi, Vietnam; ^19^Department of Infectious Diseases, Bach Mai Hospital, Hanoi, Vietnam; ^20^Department of Infectious Diseases, Tan Tock Seng Hospital, Singapore 308433

## Abstract

This study examined characteristics of HIV-infected patients in the TREAT Asia HIV Observational Database who were lost to follow-up (LTFU) from treatment and care. Time from last clinic visit to 31 March 2009 was analysed to determine the interval that best classified LTFU. Patients defined as LTFU were then categorised into permanently LTFU (never returned) and temporary LTFU (re-entered later), and these groups compared. A total of 3626 patients were included (71% male). No clinic visits for 180 days was the best-performing LTFU definition (sensitivity 90.6%, specificity 92.3%). During 7697 person-years of follow-up, 1648 episodes of LFTU were recorded (21.4 per 100-person-years). Patients LFTU were younger (*P* = 0.002), had HIV viral load ≥500 copies/mL or missing (*P* = 0.021), had shorter history of HIV infection (*P* = 0.048), and received no, single- or double-antiretroviral therapy, or a triple-drug regimen containing a protease inhibitor (*P* < 0.001). 48% of patients LTFU never returned. These patients were more likely to have low or missing haemoglobin (*P* < 0.001), missing recent HIV viral load (*P* < 0.001), negative hepatitis C test (*P* = 0.025), and previous temporary LTFU episodes (*P* < 0.001). Our analyses suggest that patients not seen at a clinic for 180 days are at high risk of permanent LTFU, and should be aggressively traced.

## 1. Introduction

Loss to followup (LTFU) in patients receiving antiretroviral therapy can cause serious consequences such as discontinuation of treatment and increased risk of death [1–3]. At a program level, LTFU can make it difficult to evaluate outcomes of treatment and care [4, 5]. In resource-limited settings, where treatment has become rapidly available following the rollout of antiretroviral therapy, LTFU presents even more challenging obstacles that require special consideration and approaches [6, 7]. 

One of the key questions in patient followup is how to define a patient as LTFU. This has varied in studies conducted in different settings [8–10]. Defining LTFU using a very early threshold, for example, a patient with no clinic visit in the last three months, may result in many patients being considered as LTFU who would return to clinic naturally at a later date. Defining LTFU with a long threshold, for example, one year, may mean delaying too long before any effort is made to track patients potentially at risk of LTFU.

The majority of research into LTFU in HIV-infected patients receiving antiretroviral treatment in resource-limited settings has been conducted in the sub-Saharan Africa region [3, 10–13]. A few studies have been conducted among Asian, mostly female, patients [14–16]. Using data from the TREAT Asia HIV Observational Database (TAHOD), this study was carried out to find the best-performing definition of LTFU and examine the characteristics of HIV-infected patients from the Asia-Pacific who were LTFU from treatment and care.

## 2. Methods

Established in 2003, TAHOD is a collaborative observational cohort study involving 18 sites in the Asia-Pacific region (see Acknowledgement). Detailed methods have been published previously [17]. Briefly, each site recruited approximately 200–300 HIV-infected patients, with recruitment based on a consecutive series of patients regularly attending a given site from a particular start-up time. Ethical approval for the study was obtained from the University of New South Wales Ethics Committee, Western Institutional Review Board, and respective local ethics committee from each TAHOD participating site.

The following data were collected: patient demographics and baseline data, CD4 and CD8 count, HIV viral load, prior and new AIDS defining illness (ADI), date and cause of death, prior and current prescribed antiretroviral treatment (ART), and reason for treatment change. Data were collected according to a common protocol. Upon recruitment, all available data prior to entry to TAHOD (considered as retrospective data) were extracted from patient case notes. Prospective data were updated six-monthly at each clinic and transferred to the data management centre for aggregation and analyses in March and September each year. TAHOD sites were encouraged to contact patients who have not been seen in the clinics in the previous 12 months.

TAHOD data submitted at March 2009 and March 2010 were used to find the best-performing definition of LTFU. TAHOD patients who had no followup after recruitment were not included in this analysis. Patients who were not seen in clinic for more than 12 months prior to the March 2010 data submission (i.e., last clinic visit prior to March 2009) were considered to be truly LTFU. The days between the last clinical visit and 31 March 2009 in the March 2009 data transfer were then used to find the interval that best classified a true LTFU in the following way. A series of cutoffs were considered, from ten to 365 days, to define patients as potentially LTFU. Each of these definitions of potential LTFU was compared with the gold standard of true LTFU, defined as no patient followup in the 12 months prior to 31 March 2010. The sensitivity and specificity of each cutoff in identifying true LTFU were calculated, and the best performing cutoff identified using the area under the receiver operator characteristic (ROC) curve. The optimal definition of LTFU identified in terms of maximising the sensitivity and specificity of true LTFU was found to be 180 days (see Results). This definition was then used in the risk factor analyses that follow.

Followup started from the last clinic visit at the March 2007 data submission. Patients who were considered LTFU before March 2007 (i.e., had no clinic visits 180 days before 31 March 2007) were excluded from the analysis. For patients enrolled after March 2007, the followup started at the time of enrolment. In terms of calculating person-years of followup, the end of followup for patients who had no clinic visit for 180 days and so were considered as LTFU was defined as 90 days after their last clinic visit. For patients not considered LTFU, the end of followup was also defined as 90 days after their last clinic visit. If a patient died, the followup was censored on the date of death if the date was within 180 days of their last clinic visit. Patients who died after March 2007 were considered to have complete followup. It should be noted that patients who were considered LTFU could return to clinic and reenter followup. The start of this reentry to followup was defined as 3 months prior to the first clinic visit that reinitiated followup. The patients that reentered followup could also be re-LTFU if the patient subsequently did not attend clinic for more than 180 days. The definitions we adopted were consistent with those in a previous study [18].

The rates of LTFU were calculated by the number of total LTFU periods divided by the total duration of followup contributed by the patients included in the analysis [18]. Because of the reentering and re-LTFU, patients could contribute more than one episode of LTFU in this analysis. The rates were further calculated in different strata, including age, sex, exposure category, hepatitis B and C infection, year since HIV infection, calendar year, the latest CD4 count and viral load, antiretroviral treatment status, CDC disease stages, prophylaxis (coded as receiving or not), and haemoglobin level, all taken at the start of each episode.

Factors associated with LTFU were assessed by multivariate Poisson regression models, using generalised estimating equations, to allow for multiple events of LTFU in the same patients. CD4 count, HIV viral load, antiretroviral treatment, AIDS diagnosis, and haemoglobin tests were included as time-dependent variables and updated at the time the new measurement or diagnosis was available.

Patients who had at least one episode of LTFU were then categorised into two groups: those who had no more clinical visits in the database (permanently LTFU) and those who later reentered followup (temporary LTFU). Multivariate logistic regression models were used to compare the characteristics in patients who were considered permanently LTFU with those who were temporary LTFU. All covariates were taken at the end of the episode in patients with truly LTFU or at the end of the first episode in patients considered temporary LTFU.

Multivariate models were built using a forward-stepwise approach. The final model included covariates that remained significant at the *P* < 0.05 level. Nonsignificant variables were also presented and adjusted for the final multivariate models. Data management and statistical analyses were performed using SAS for Windows (SAS Institute Inc., Cary, NC, USA) and Stata (StataCorp, STATA 10.1 for Windows, College Station, TX, USA).

## 3. Results

In March 2007, there were 2565 patients in the database. 1061 patients were subsequently enrolled in TAHOD up to March 2010. A total of 3626 patients from TAHOD who had follow-up visits in the clinic were included in this analysis. During the study period (from March 2007 to March 2010), there were 54 patients who died and considered to have complete followup.

Using days between last clinic visit and 31 March 2009 in the March 2009 data transfer, we identified the interval that best classifies a true LTFU (i.e., no clinic visit after 31 March 2009). An interval of 180 days was determined as the best-performing definition (Table 1, sensitivity 90.6%, specificity 92.3%). Using 180 days as the LTFU cutoff, during 7697 person-years of followup, a total of 1648 episodes of LTFU from 1298 patients were identified, giving a crude LTFU rate of 21.4 per 100 person-years (95% confidence interval, CI, 20.4 to 22.5). Of those 1648 episodes of LTFU identified using 180 days as the cutoff, 48% were considered permanently LTFU (i.e., the patient did not return to clinic before 31 March 2010), corresponding to 45% of the 1298 patients.

The patient characteristics are summarised in Table 2. The majority of patients were male (71%), aged between 36 and 45 years (40%), and reported heterosexual transmission (64%). Chinese (27%), Thai (26%), and Indian (11%) were the main ethnic groups. At recruitment, approximately 12% did not have a CD4 count test, and of those tested, the majority had a CD4 count more than 200 cells/*μ*L. Nearly half (45%) did not have an HIV viral load test, and of those tested, the majority were below 500 copies/mL. Close to half of the patients (46%) were diagnosed with an AIDS defining illness at recruitment, with tuberculosis being the main illness. Most patients (63%) had been reported to be diagnosed with HIV for less than 6 years when recruited to TAHOD (measured as the time from first reported positive HIV test). Less than 10% of the patients were coinfected with either hepatitis B or hepatitis C. At recruitment, the majority of patients had normal haemoglobin level. At the start of study followup, most of the patients were on antiretroviral therapy including three or more drugs in combination including at least one nucleoside reverse transcriptase inhibitor (NRTI) and one nonnucleoside reverse transcriptase inhibitor. Over 20% of patients were in a combination with at least one NRTI and a protease inhibitor (PI). All patients were receiving, or started, antiretroviral therapy during followup. 

Table 3 summarises univariate and multivariate analyses of factors associated with LTFU using 180 days as cut-off. In univariate analyses, the rate of LTFU was significantly lower in patients with a current CD4 counts above 200 cells/*μ*L compared to patients with a CD4 count less than 100 cells/*μ*L, but this was not significant in the final multivariate model. In the final multivariate model (Table 3), factors associated with LTFU included age (younger patients had higher rate of LTFU), current HIV viral load (either patients with HIV viral load ≥500 copies/mL or no tests in recent 180 days had higher rate of LTFU), history of HIV infection (patients with shorter history of HIV infection had higher rate of LTFU), hepatitis C infection (patients with positive hepatitis C antibody had higher rate of LTFU), and, finally, current combination of antiretroviral treatment (compared to patients on triple-drug regimen with at least one NRTI and one NNRTI, patients receiving no-, single-, or double-drug antiretroviral therapy, or a triple-drug regimen containing at least one NRTI and one PI, had higher rate of LTFU).

Table 4 shows factors that predict permanent LTFU among patients who had no clinic visit for 180 days and so met our optimal definition of LTFU. In the final multivariate model, patients permanently LTFU were more likely to be older, have not been anemic, have no recent HIV viral load test, have tested negative for hepatitis C infection or have never tested for hepatitis C, and have had more than one episode of previous temporary LTFU.

## 4. Discussion

We found that an interval of 180 days between clinic visits was the best-performing definition of LTFU based on sensitivity and specificity in identifying true LTFU. By this definition, we observed that approximately one in five patients in our cohort would miss clinic visits for more than 180 days and so become defined as LTFU. Among these patients in our cohort close to half eventually returned to followup, with half becoming truly lost to HIV-related treatment and care.

The 180-day cutoff has been used by other studies as a working definition of LTFU [10, 19–21]. Other intervals have also been proposed as measurements of classifications of LTFU, such as 90 days [8] and 365 days [9]. Regional- and cohort-dependent characteristics, such as scheduled clinic visits, patient burden, and drug availability could result in specific intervals that best categorise patients at risk of LTFU. Nevertheless, a 180-day (or 6-month) cutoff is an appealing and easy-to-apply definition that could be used in different clinical settings in the Asia-Pacific region to flag patients at risk of being permanently lost to treatment and care. Our analyses suggest patients with no clinic visits for six months are at high risk of being permanently lost and should be aggressively traced.

 Chi et al. also found that a cutoff of 180 days was optimal to define LTFU after analysing data from the Africa, Asia, and Latin America regions of the IeDEA collaboration (including data from our cohort) [22]. There are some methodological differences between our analyses, principally regarding minimum numbers of patients for site inclusion. Chi et al. found quite extensive heterogeneity between sites, something we also found to a lesser extent. However, it is nevertheless reassuring that we found a similar optimal cutoff of 180 days without clinic visits to define LTFU. With rapid scaling up of antiretroviral treatment taking place globally, there is a need to adopt a universal consistent definition of LTFU, or a general algorithm to define cutoffs, to evaluate HIV treatment programs in different regions [6, 7, 19].

Over one in five patients in our cohort failed to come to clinic for more than 180 days in a given year. Similar rates have also been found in patients from Africa [3, 11]. However, the LTFU rate was lower in EuroSIDA [23], a large prospective cohort study with HIV-infected patients mainly from Europe (using one year as a cutoff). Approximately half of the patients who experienced LTFU in our study later came back to clinic, and patients who had a previous episode of LTFU were more likely to prove to be true LTFU, similar to previous findings [18].

We found that younger patients, patients infected with hepatitis C, and patients with detectable or unmeasured viral load were more likely to experience LTFU. These findings are all consistent with previous study findings [10, 11, 24–26]. Patients with undetectable viral load are likely to be motivated and adherent to antiretroviral treatment and thus remain in care. Among those patients who experienced LTFU, we found that those who tested negative for hepatitis C infection or were never tested for hepatitis C were more likely to be permanently LTFU. This finding seems counterintuitive, but it might be that patients who have tested positive for hepatitis C receive more medical attention from their clinicians and thus prove less likely to be permanently LTFU. Among patients identified as LTFU, anemic patients were also more likely to be permanently lost to treatment and care. Anemia has been shown to be a strong prognostic marker for HIV disease progression and survival [27], which could, at least in part, explain these patients failing to return to followup.

Compared to patients on NNRTI-based regimen, patients receiving no-, single-, or double-drug antiretroviral therapy or a triple-drug regimen containing PI were more likely to experience LTFU. The reasons for this are not clear. The greater loss to followup may be associated with increased drug toxicity, either resulting in a patient receiving mono- or dual therapy or from receiving a PI. Patients receiving PI-based regimens are also those who are more likely to be on a second line regimen, a regimen that may be substantially more expensive than first line. In the Asia Pacific region, out-of-pocket expenses are needed to pay for treatment in some clinics. Hence, the lost to followup may be associated with drug availability or affordability. It is worth noting that patients receiving mono- or dual therapy, or a PI based regimen, were also associated with being less likely to be permanently lost to followup, that is to say more likely to return to clinic (albeit not quite statistically significantly so). This possibly supports the idea of these regimens being associated with short-term drug availability or affordability issues. Unfortunately, data are not available to address this issue in any greater detail.

It has been shown that, in resource-limited settings, predominantly in Africa, patients who are LTFU have a much poorer prognosis than patients who remain in followup [5]. In part, this is due to a proportion of patients who die not having vital status information updated at their treatment site. The extent to which this occurs in TAHOD is uncertain. While it seems likely that at least some patients who are LTFU have died without this information reaching the site, the lack of association between key measures of HIV disease progression, such as CD4 count and AIDS defining illnesses, and LFTU suggests it may be lower than in African settings. However, this association between LTFU and poorer prognosis underpins the need for consistent definitions of LTFU in research cohort studies, and where there are possible active patient tracing strategies or at least sampling-based approaches [28] to ensure comparability of results across studies and settings.

Several limitations should be considered in interpreting the results in this paper. First, TAHOD participating sites are generally urban referral centres, and the patients recruited in TAHOD were those regularly attending a given TAHOD site. Hence, TAHOD patients are not representative of all HIV-infected patients in the Asia and Pacific region. The overall rate of LTFU we saw in our study is therefore likely to be an underestimate of rates across the region. However, the effect of these sampling biases on the optimal definition of LTFU and on the covariate analyses is arguably less strong. It is reassuring that our estimate of the optimal definition of LTFU is consistent with that seen across Africa and Latin America [22]. Second, since antiretroviral treatment has become more decentralised and available in distant or rural communities with rapid scale-up programs, patients might choose to receive treatment and care locally rather than at tertiary and referral centres [29, 30]. Consequently, patients may have been retained in care but not necessarily in the clinics involved in this study. Information on referral to other health facility was only recently included in the data collection, so we could not further verify if patients were retained in care or truly loss to health services. Third, we do not collect data on the measures TAHOD sites undertake to routinely trace patients who are LTFU. These measures differ across sites according to local practices and conditions. Effective patient tracking and recording are essential to program evaluation and maintenance of treatment and care [1, 18]. What patient tracking measures are effective in retaining patients in treatment and care in the Asia-Pacific region is an area that deserves further research. We also do not have data on transportation [31], social and economic status [32], pregnancy for women [10], and community support [33], all of which have been found to be important determinants of LTFU. Lastly, the patients included in this study were all receiving, or started, antiretroviral treatment and had clinical assessments. Consequently, the results cannot be extrapolated to patients not yet initiated on antiretroviral therapy. Research into followup among HIV-infected patients not receiving antiretroviral treatment in the Asia-Pacific region needs to be considered [34–36], particularly in the context of the move to start treatment earlier.

## 5. Conclusion

With rapid scaleup of antiretroviral treatment, it is essential to study factors that predict loss to followup and identify patients at risk of loss to treatment and care, particularly in resource-limited settings. At the treatment and care level, this can maintain efficacy of antiretroviral therapy and avoid adverse events. At the program evaluation level, the impact of loss to followup on overall treatment outcome, disease progression, and survival can then be accounted for with appropriate statistical adjustments. Collaboration with HIV treatment programs in other regions in studies on LTFU and in particular standardisation of LTFU definitions are essential for reporting and program evaluation.

## Figures and Tables

**Table 1:** Receiver operating characteristic (ROC) analysis for the best-performing definition for loss to followup.

Cutoff (days)	Sensitivity (%)	Specificity (%)	Area under ROC	Cutoff (days)	Sensitivity (%)	Specificity (%)	Area under ROC
10	99.67	16.97	58.32	160	90.96	90.77	90.87
20	99.02	24.32	61.67	170	90.64	91.44	91.04
30	98.05	31.31	64.68	175	90.64	92.05	91.34
40	96.82	39.90	68.36	**180 **	**90.55 **	**92.26 **	**91.41 **
50	96.34	49.52	72.93	185	90.23	92.53	91.38
60	95.77	57.20	76.48	190	89.33	93.01	91.17
70	95.28	65.52	80.40	200	88.52	93.44	90.98
80	95.11	71.26	83.19	210	87.79	94.13	90.96
90	94.71	77.62	86.16	240	85.26	95.25	90.26
100	94.22	80.91	87.57	270	83.55	96.43	89.99
120	93.24	86.18	89.71	300	82.00	97.04	89.52
150	91.53	90.17	90.85	365	78.99	97.73	88.36

True LTFU defined as no patient followup in the 12 month prior to 31 March 2010. Each cutoff used as a potential definition of LTFU was the days between last clinical visit and 31 March 2009 in the March 2009 data transfer. The sensitivity and specificity of each cutoff in identifying true LTFU were calculated, and the optimal cutoff identified based on ROC analysis.

**Table 2:** Patient characteristics.

Total	3626	
Characteristics	Number	%
Sex		
Male	2567	71
Female	1059	29

Current age (years)		
≤35	1383	38
36–45	1449	40
46+	794	22

Reported exposure		
Heterosexual contact	2337	64
Homosexual contact	749	21
Injecting drug use	263	7
Other/unknown	277	8

Ethnicity		
Chinese	989	27
Indian	390	11
Thai	933	26
Other/unknown	1314	36

Baseline CD4 count (cells/*μ*l.)		
≤100	239	7
101–200	406	11
201+	2531	70
Missing	450	12

Baseline HIV RNA (copies/ml)		
≤500	1482	41
501+	379	10
Missing	1765	49

CDC disease stage at baseline		
Stage A	1621	45
Stage B	321	9
Stage C	1684	46

Tuberculosis diagnosis at baseline		
No	2758	76
Yes	868	24

Time since HIV infection (years)		
≤5	2295	63
6+	1246	34
Missing	85	2

Hepatitis B infection		
No	2297	63
Yes	257	7
Not tested	1072	30

Hepatitis C infection		
No	2007	55
Yes	324	9
Not tested	1295	36

Anemia at baseline		
No	2480	68
Yes	597	16
Haemoglobin not tested	567	16
Antiretroviral treatment at baseline		
3 + (NRTI + NNRTI)	2224	61
3 + (NRTI + PI)	744	21
No/mono/double drug	583	16
3 + (other combination)	75	2

Anemia: haemoglobin <13 g/dl (male), <11 g/dl (female); NRTI: nucleoside reverse transcriptase inhibitor; NNRTI: nonnucleoside reverse transcriptase inhibitor; PI: protease inhibitor.

**Table 3:** Factors associated with permanent or temporary LTFU, defined as no clinic visit for 180 days, among all patients under followup.

	Person- years	Number LTFU	Crude Rate^1^	Adjusted						
				95% CI	IRR^2^	95% CI	*P* value	IRR^2^	95% CI	*P* value
Sex										
Male	5468.1	1206	22.06	(20.85, 23.34)	1.00			1.00		
Female	2229.2	442	19.83	(18.06, 21.77)	1.10	(0.98, 1.24)	0.090	1.04	(0.93, 1.17)	0.446

Current age (years)										
≤35	2210.4	575	26.01	(23.97, 28.23)	1.00			1.00		0.002^3^
36*∼*45	3320.2	718	21.62	(20.10, 23.27)	0.82	(0.74, 0.92)	0.001	0.89	(0.79, 1.00)	0.050
46+	2166.6	355	16.39	(14.77, 18.18)	0.69	(0.60, 0.79)	<0.001	0.76	(0.66, 0.88)	<0.001

Reported exposure										
Heterosexual contact	5144.5	985	19.15	(17.99, 20.38)	1.00			1.00		
Homosexual contact	1707.2	344	20.15	(18.13, 22.40)	1.10	(0.93, 1.29)	0.275	1.05	(0.89, 1.25)	0.540
Injecting drug use	344.3	125	36.31	(30.47, 43.27)	1.21	(0.97, 1.51)	0.098	1.10	(0.86, 1.40)	0.437
Other/unknown	501.3	194	38.70	(33.62,44.55)	1.64	(1.37, 1.98)	<0.001	1.56	(1.29, 1.88)	<0.001

Current CD4 count (cells/*μ*l.)										
≤100	233.7	69	29.52	(23.32, 37.38)	1.00			1.00		
101–200	635.7	136	21.40	(18.09, 25.31)	0.92	(0.68, 1.22)	0.551	0.96	(0.72, 1.29)	0.800
201+	6327.6	1181	18.66	(17.63, 19.76)	0.75	(0.58, 0.96)	0.023	0.79	(0.61, 1.02)	0.071
Missing	500.3	262	52.37	(46.40, 59.11)	1.18	(0.90, 1.55)	0.235	0.99	(0.74, 1.31)	0.922

Current HIV RNA (copies/ml)										
≤500	4213.7	679	16.11	(14.95, 17.37)	1.00			1.00		0.021^3^
501+	537.1	158	29.42	(25.17, 34.38)	1.71	(1.43, 2.04)	<0.001	1.24	(1.03, 1.51)	0.026
Missing	2946.4	811	27.52	(25.69, 29.49)	1.75	(1.55, 1.98)	<0.001	1.64	(1.45, 1.86)	<0.001

CDC disease stage										
Stage A	3205.1	828	25.83	(24.13, 27.65)	1.00			1.00		
Stage B	801.6	118	14.72	(12.29, 17.63)	0.93	(0.76, 1.14)	0.507	0.95	(0.77, 1.17)	0.623
Stage C	3690.5	702	19.02	(17.67, 20.48)	0.84	(0.75, 0.93)	0.001	0.92	(0.82, 1.02)	0.125

Tuberculosis diagnosis										
Yes	1806.7	372	20.59	(18.60, 22.79)	1.00			1.00		
No	5890.6	1276	21.66	(20.51, 22.88)	1.04	(0.92, 1.18)	0.537	0.98	(0.87, 1.12)	0.801

Time since HIV infection (years)										
≤5	3477.2	785	22.58	(21.05, 24.21)	1.00			1.00		0.005^3^
6+	4115.7	844	20.51	(19.17, 21.94)	0.84	(0.75, 0.94)	0.002	0.89	(0.79, 1.00)	0.048
Missing	104.3	19	18.21	(11.61, 28.55)	0.58	(0.36, 0.94)	0.027	0.49	(0.30, 0.79)	0.004

Hepatitis B infection										
Yes	584.5	112	19.16	(15.92, 23.06)	1.00			1.00		
No	5101.9	883	17.31	(16.20, 18.49)	0.93	(0.76, 1.13)	0.474	0.90	(0.74, 1.10)	0.319
N/A	2010.8	653	32.48	(30.08, 35.06)	0.98	(0.80, 1.21)	0.859	1.07	(0.85, 1.35)	0.548

Hepatitis C infection										
Yes	541.4	149	27.52	(23.44, 32.31)	1.00			1.00		0.030^3^
No	4692.8	796	16.96	(15.82, 18.18)	0.81	(0.67, 0.98)	0.029	0.81	(0.67, 0.98)	0.034
N/A	2463.0	703	28.54	(26.51, 30.73)	0.75	(0.62, 0.91)	0.004	0.77	(0.63, 0.93)	0.008
Current anemia (male < 13 g/dl, female < 11 g/dl)										
Yes	1021.1	155	15.18	(12.97, 17.77)	1.00			1.00		
No	5771.6	1157	20.05	(18.92, 21.24)	1.09	(0.92, 1.30)	0.302	1.11	(0.94, 1.32)	0.227
N/A	904.5	336	37.15	(33.38, 41.34)	1.31	(1.07, 1.59)	0.008	1.09	(0.89, 1.34)	0.382
Current ART^4^										
3 + (NRTNRTI)	4830.8	942	19.50	(18.29, 20.79)	1.00			1.00		0.001^3^
3 + (NRTI + PI)	1898.3	377	19.86	(17.95, 21.97)	1.21	(1.06, 1.38)	0.005	1.22	(1.07, 1.39)	0.003
No/mono/double ARV	762.7	300	39.33	(35.12, 44.05)	2.18	(1.90,2.50)	<0.001	1.92	(1.66, 2.22)	<0.001
3 + (other combination)	205.4	29	14.12	(9.81, 20.32)	0.95	(0.65, 1.38)	0.786	1.01	(0.69, 1.47)	0.975

(1) Crude rate, per 100 person-years.

(2) Stratified by TAHOD sites.

(3) Overall for test for trend (ordinal categorical covariates) or for homogeneity (nominal categorical covariates).

(4) ART: NRTI: nucleoside reverse transcriptase inhibitor; NNRTI: nonnucleoside reverse transcriptase inhibitor; PI: protease inhibitor.

**Table 4:** Factors that predict permanent LTFU in patients without a clinic visit for 180 days.

	Number	True loss	%	OR^1^	95% CI	*P* value	Adjusted OR^1^	95% CI	*P* value
Sex									
Male	1206	584	48.4	1.00			1.00		
Female	442	209	47.3	0.89	(0.69, 1.15)	0.359	0.80	(0.61, 1.05)	0.104

Current age (years)									
≤35	568	278	48.9	1.00			1.00		0.097^2^
36*∼*45	717	340	47.4	1.33	(1.03, 1.71)	0.031	1.31	(1.00, 1.72)	0.050
46+	363	175	48.2	1.27	(0.94, 1.72)	0.118	1.28	(0.93, 1.77)	0.128

Reported exposure									
Heterosexual contact	985	443	45.0	1.00			1.00		
Homosexual contact	344	199	57.8	1.12	(0.78, 1.60)	0.532	1.24	(0.85,1.81)	0.262
Injecting drug use	125	55	44.0	1.01	(0.59, 1.73)	0.969	1.32	(0.72, 2.41)	0.364
Other/unknown	194	96	49.5	1.07	(0.69, 1.64)	0.773	1.22	(0.78, 1.93)	0.382

Current CD4 count (cells/*μ*l.)									
≤100	58	36	62.1	1.00			1.00		
101–200	129	66	51.2	0.76	(0.36, 1.60)	0.471	0.99	(0.47, 2.13)	0.989
201+	1068	465	43.5	0.62	(0.33, 1.18)	0.144	0.82	(0.42, 1.59)	0.551
Missing	393	226	57.5	1.50	(0.77, 2.93)	0.238	1.18	(0.58, 2.42)	0.649

Current HIV RNA (copies/mL)									
≤500	598	230	38.5	1.00			1.00		0.011^2^
501+	153	78	51.0	1.02	(0.68, 1.52)	0.924	0.94	(0.62, 1.42)	0.767
Missing	897	485	54.1	2.13	(1.63, 2.80)	<0.001	1.54	(1.13, 2.09)	0.006

CDC disease stage									
Stage A	828	413	49.9	1.00			1.00		
Stage B	121	54	44.6	0.77	(0.48, 1.22)	0.258	0.70	(0.43, 1.14)	0.154
Stage C	699	326	46.6	1.00	(0.78, 1.27)	0.975	1.05	(0.81, 1.36)	0.702

Tuberculosis diagnosis									
Yes	361	186	51.5	1.00			1.00		
No	1287	607	47.2	0.87	(0.66, 1.16)	0.342	0.85	(0.63, 1.15)	0.297

Time since HIV infection (years)									
≤5	771	400	51.9	1.00			1.00		
6+	858	389	45.3	1.25	(0.98, 1.60)	0.076	1.03	(0.79, 1.34)	0.835
Missing	19	4	21.1	0.37	(0.12, 1.17)	0.091	0.43	(0.13, 1.43)	0.170

Hepatitis B infection									
Yes	112	47	42.0	1.00			1.00		
No	883	431	48.8	1.30	(0.84, 2.03)	0.243	1.35	(0.84, 2.16)	0.222
N/A	653	315	48.2	1.31	(0.82, 2.09)	0.253	1.03	(0.60,1.76)	0.908
Hepatitis C infection									
Yes	149	66	44.3	1.00			1.00		0.004^2^
No	796	376	47.2	1.57	(1.01, 2.45)	0.046	1.66	(1.04, 2.66)	0.034
N/A	703	351	49.9	1.96	(1.26, 3.05)	0.003	2.16	(1.35, 3.46)	0.001

Current anemia (male < 13 g/dL, female < 11 g/dL)									
Yes	141	87	61.7	1.00			1.00		<0.001^2^
No	1065	456	42.8	0.53	(0.35, 0.81)	0.003	0.50	(0.32, 0.76)	0.001
N/A	442	250	56.6	1.15	(0.73, 1.81)	0.549	0.78	(0.49, 1.26)	0.310

Current ART**									
3 + (NRTI + NNRTI)	911	404	44.3	1.00			1.00		
3 + (NRTI + PI)	356	167	46.9	0.76	(0.57, 1.02)	0.072	0.74	(0.54,1.01)	0.057
No/mono/double ARV	352	209	59.4	0.93	(0.69, 1.26)	0.644	0.78	(0.57, 1.08)	0.137
3 + (other combination)	29	13	44.8	0.89	(0.40, 1.98)	0.770	0.85	(0.38, 1.94)	0.707
Previous episode of temporary LTFU									
None	1298	589	45.4	1.00			1.00		<0.001^2^
Once	296	158	53.4	2.79	(2.05, 3.80)	<0.001	2.71	(1.97, 3.72)	<0.001
Twice	54	46	85.2	31.76	(13.91, 72.52)	<0.001	27.75	(12.03, 64.01)	<0.001

(1) Stratified by TAHOD sites.

(2) Overall for test for trend (ordinal categorical covariates) or for homogeneity (nominal categorical covariates).

(3) ART: NRTI: nucleoside reverse transcriptase inhibitor; NNRTI: nonnucleoside reverse transcriptase inhibitor; PI: protease inhibitor.
